# NFAT5 Is Involved in GRP-Enhanced Secretion of GLP-1 by Sodium

**DOI:** 10.3390/ijms22083951

**Published:** 2021-04-12

**Authors:** Caini Fan, Laureano D. Asico, Van Anthony M. Villar, Jessica Hunt, Santiago Cuevas, Ines Armando, Pedro A. Jose, Prasad R. Konkalmatt

**Affiliations:** 1 Division of Renal Diseases & Hypertension, The George Washington University School of Medicine & Health Science, Washington, DC 20052, USA; fcn0067@126.com (C.F.); lasico@gwu.edu (L.D.A.); vvillar@gwu.edu (V.A.M.V.); jessicahunt@gwu.edu (J.H.); scg76@hotmail.com (S.C.); iarmando@gwu.edu (I.A.); pjose@mfa.gwu.edu (P.A.J.); 2 Department of Hypertension, Henan Provincial People’s Hospital, Zhengzhou University People’s Hospital, Henan University People’s Hospital, Zhengzhou 450003, China; 3 Department of Physiology/Pharmacology, The George Washington University School of Medicine & Health Science, Washington, DC 20052, USA

**Keywords:** gastrin, gastrin-releasing peptide, glucagon-like peptide-1, NFAT5

## Abstract

Gastrin, secreted by G-cells, and glucagon-like peptide-1 (GLP-1), secreted by L-cells, may participate in the regulation of sodium balance. We studied the effect of sodium in mice in vivo and mouse ileum and human L-cells, on GLP-1 secretion, and the role of NFAT5 and gastrin-releasing peptide receptor (GRPR) in this process. A high-sodium diet increases serum GLP-1 levels in mice. Increasing sodium concentration stimulates GLP-1 secretion from mouse ileum and L-cells. GRP enhances the high sodium-induced increase in GLP-1 secretion. High sodium increases cellular GLP-1 expression, while low and high sodium concentrations increase NFAT5 and GRPR expression. Silencing NFAT5 in L-cells abrogates the stimulatory effect of GRP on the high sodium-induced GLP-1 secretion and protein expression, and the sodium-induced increase in GRPR expression. GLP-1 and gastrin decrease the expression of Na^+^-K^+^/ATPase and increase the phosphorylation of sodium/hydrogen exchanger type 3 (NHE3) in human renal proximal tubule cells (hRPTCs). This study gives a new perspective on the mechanisms of GLP-1 secretion, especially that engendered by ingested sodium, and the ability of GLP-1, with gastrin, to decrease Na^+^-K^+^/ATPase expression and NHE3 function in hRPTCs. These results may contribute to the better utilization of current and future GLP-1-based drugs in the treatment of hypertension.

## 1. Introduction

Increased sodium intake is one of the most important factors in the pathogenesis of hypertension. Normally, after a high sodium diet intake, the body excretes the excess sodium through a variety of mechanisms to maintain sodium and fluid balance and keep the blood pressure in the normal range [1]. However, in salt-sensitive states, a high sodium intake results in sodium accumulation in the body due to impaired renal sodium excretion, related to the failure of pressure-natriuresis and compensatory neural and hormonal mechanisms; consequently, the blood pressure increases. In salt-resistant states, sodium is excreted efficiently to maintain the blood pressure in the normal range [2]. The gastrointestinal-renal axis plays an important role in the regulation of fluid and electrolyte balance and blood pressure [3,4,5]. Sensing the amount of ingested sodium by the gastrointestinal tract (GIT) may be an important mechanism by which sodium balance is regulated [3,4,5]. Gastrin, secreted by G-cells in the stomach and duodenum, is involved in the regulation of renal sodium excretion [3,4,5]. Glucagon-like peptide-1 (GLP-1) is another intestinal hormone that may also participate in the regulation of sodium balance and blood pressure [5,6,7].

Ingested nutrients, e.g., fat, carbohydrates, glucose, fructose, protein, amino acids, and minerals (e.g., sodium), stimulate intestinal GLP-1 secretion [5,6,7,8]. Gastrin releasing peptide (GRP) may mediate the increase in GLP-1 secretion, because the intravenous injection of a G protein-coupled receptor (GRPR) antagonist, BW10, prevented the increase in GLP-1 secretion in distal intestines induced by fat placed in the duodenum [9]. Moreover, the increase in GLP-1 secretion in response to glucose, given by gastric gavage, is impaired in GRPR-deficient mice [10]. The oral administration of water, sodium chloride, or glucose in rats, and sodium chloride or glucose in humans, increases plasma GLP-1 levels [5,6,7,8,9,10,11]. GRP can increase sodium entry into Swiss 3T3 cells by increasing Na^+^/H^+^ exchanger (NHE) activity, which may be involved in the stimulation of GLP-1 secretion [12]. The ability of nonmetabolizable sugars to increase GLP-1 secretion by the ileum is sodium-dependent, involving sodium-glucose cotransporters [13]. Salt (NaCl) intake can also regulate serum GLP-1 levels in normotensive salt-sensitive humans [14]. However, the effect of varying sodium intake and concentrations on GLP-1 secretion and potential mechanisms remain to be elucidated.

NFAT5 (aka TonEBP/OREBP) is a Rel-family transcription factor involved in protecting cells from hypertonic stress [15]. However, NFAT5 can also be regulated by stimuli independent of tonicity, including glucose, calcium, and sodium chloride/superoxide [15,16,17]. Gastrin transcription in human G-cells can be stimulated by sodium [18]. Putative salt-inducible transcription factor NFAT5 binding consensus sequences (5′-TGGAAANYNY-3′ or 5′-RNRNTTTCCA-3′) in the GRPR promoter are −865 nucleotides upstream of the transcription start site (https://epd.epfl.ch//index.php; *p* = 0.0001, accessed on 17 July 2019). We hypothesized that NFAT5 and GRPR may be involved in the GLP-1 secretion in response to sodium concentration. Therefore, we determined the effect of varying amounts of sodium intake on GLP-1 secretion in mice and involvement of NFAT5 and GRPR in GLP-1 secretion or expression in response to varying concentrations of sodium in human L-cells and mouse ileum.

## 2. Results

### 2.1. Effect of Increasing Sodium Content in the Food on GLP-1 Secretion In Vivo

**Figure 1:**
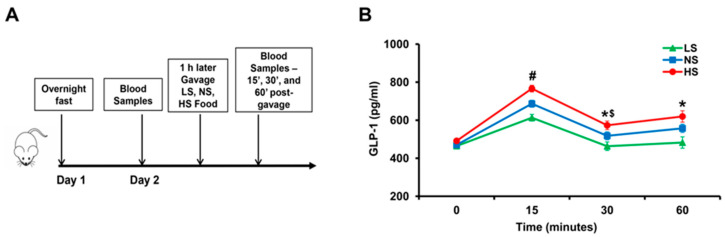
The effect of varying amounts of sodium in the diet on serum GLP-1 levels in BALB/c mice. (**A**) After an overnight fast, on the morning of day 2, 120 μL of blood samples were drawn from the caudal vein. One hour later, the mice were gavaged with 1 mL of low salt (LS), normal salt (NS), or high salt (HS) food which contained <0.04 mmol sodium, 0.56 mmol sodium, and 0.84 mmol sodium/0.1 kg body weight, respectively. Then 120 μL of blood were drawn at 15’, 30’, 60’ post gavage. (**B**) Serum GLP-1 in BABL/c mice at different time points after gavage. *^#^ p* < 0.05 vs. NS or LS at 15’, * p < 0.05 vs. LS at 30’ and 60’, ^$^ *p* < 0.05 vs. HS at 15’ or 60’. One-way ANOVA, Holm–Sidak test, *n* = 3–6/group.

To determine the effect of varying amounts of sodium in the food on GLP-1 secretion, we gavaged salt-resistant BALB/c mice with 1 mL of food containing different amounts of sodium; low salt (LS), normal salt (NS), or high salt (HS) food. LS, NS, and HS diet contained <0.04 mmol sodium, 0.56 mmol sodium, and 0.84 mmol sodium/0.1 kg body weight, respectively. Serum GLP-1 levels were measured before and after gavage (Figure 1A). Serum GLP-1 levels increased in all mice (*p* < 0.05), 15′ after gavage (Figure 1B). GLP-1 levels were highest in mice gavaged with HS, followed by mice gavaged with NS, and least in mice gavaged with LS (*p* < 0.05). GLP-1 levels decreased 30’ after gavage in all groups compared with 15’ but remained higher than 0 time in mice gavaged with HS and NS (*p* < 0.05) but not in mice gavaged with LS. GLP-1 levels at 1 h after gavage remained higher than before gavage in the NS and HS groups. These results show that GLP-1 secretion into the blood is influenced by the sodium content of food and suggests that GLP-1 may be an effector enterokine that responds to an increase in sodium intake.

### 2.2. Gastrin Releasing Peptide (GRP) Enhances Sodium-Induced Increase in GLP-1 Secretion by Human L-Cells

**Figure 2:**
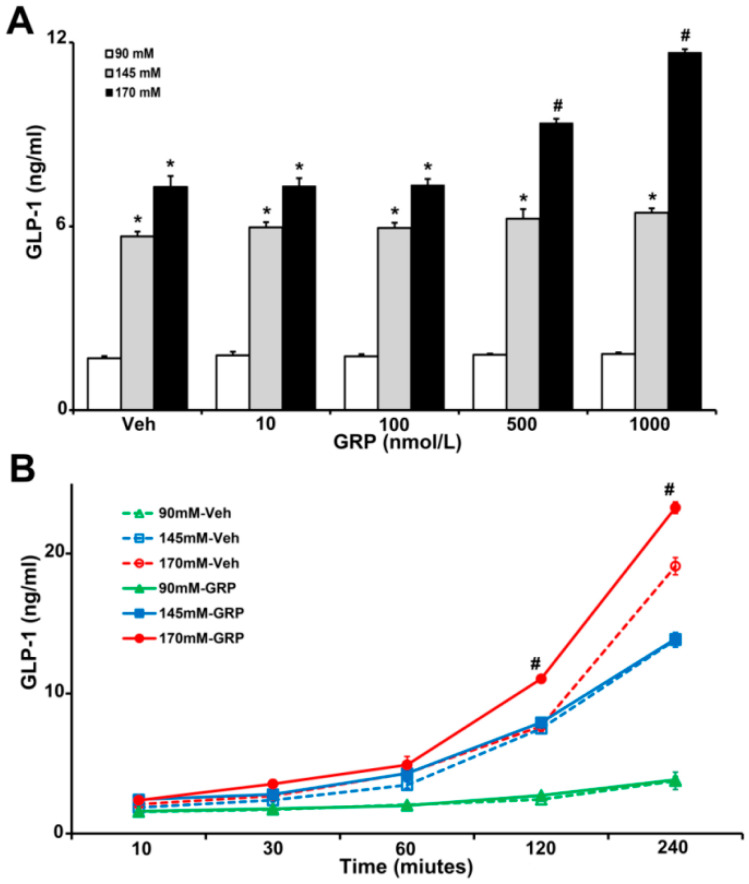
The effect of varying concentrations sodium (NaCl) and GRP on GLP-1 secretion in L-cells. The same quantity of cells were exposed to 90 mM, 145 mM, or 170 mM NaCl, and (**A**), treated with varying concentrations of GRP (10, 100, 500, and 1000 nmol/L) for 4 h, or (**B**), treated with 1000 nmol/L GRP for 30-, 60-, 120-, and 240-min. Vehicle (Veh) served as control. * *p* < 0.05 vs. 90 mM, ***^#^*** *p* < 0.05 vs. all others, one-way ANOVA, Holm–Sidak test, *n* = 3/treatment.

Gastrin-releasing-peptide (GRP) is a potent GLP-1 secretagogue both in vitro and in vivo and is especially important in the initial rapid rise in GLP-1 secretion after a meal [9]. To show the relevance of our studies in humans, we used human L-cells, which secretes GLP-1 [19], to determine the effect of sodium and GRP on GLP-1 secretion. We incubated the same number of L-cells in media containing increasing concentrations of NaCl (90 mM, 145 mM, and 170 mM), in the presence of vehicle or GRP (10, 100, 500, and 1000 nM) for 4 h. As shown in Figure 2A, GLP-1 secretion in L-cells incubated with 145 mM and 170 mM NaCl in the medium was 3~7-fold higher than those incubated with 90 mM NaCl with or without GRP. Co-treatment with 170 mM NaCl and 500 nM or 1000 nM GRP further increased GLP-1 secretion (1.3-fold or 1.6-fold, respectively). This effect was not observed in L-cells treated with 90 mM or 145 mM NaCl (Figure 2A).

We next determined the time course of the GLP-1 secretion in L-cells, in response to GRP. We incubated L-cells in 90 mM, 145 mM, and 170 mM NaCl medium in the presence of vehicle or 1000 nM GRP for 10 min, 30 min, 1 h, 2 h, and 4 h. As shown in Figure 2B, GLP-1 secretions increased with time in L-cells incubated in 145 mM and 170 mM NaCl and were higher than in L-cells incubated in 90 mM NaCl. However, in the presence of GRP only L-cells incubated in 170 mM NaCl medium secreted more GLP-1; this effect started at 2 h and lasted up to 4 h. At the latest time point (4 h), cells incubated with 145 mM NaCl, alone, or with GRP, also secreted more GLP-1. However, there was no additional effect of GRP, which was the case with 170 mM NaCl.

**Figure 3:**
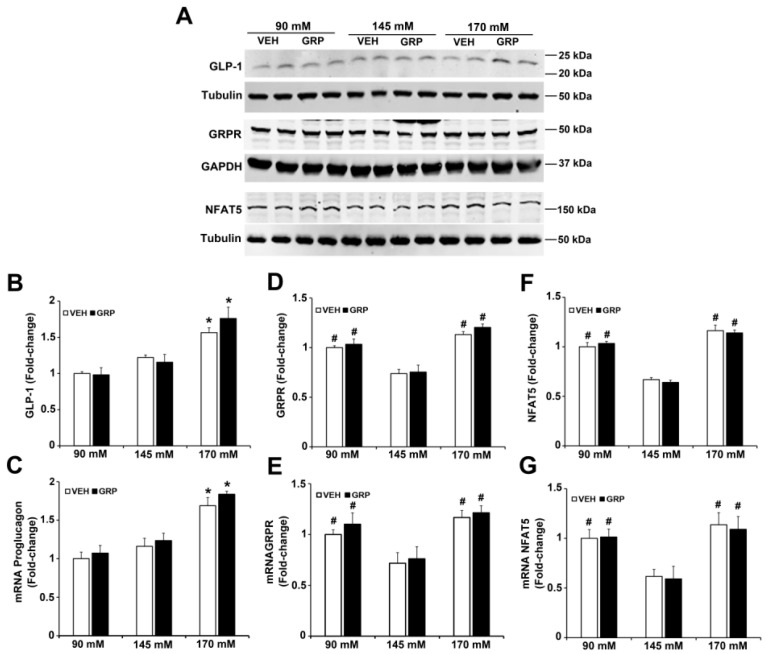
Expressions of GLP-1, GRPR, and NFAT5 in L-cells exposed to varying NaCl concentrations with or without GRP treatment. The same quantities of L-cells were exposed to 90 mM, 145 mM, or 170 mM NaCl, and treated with vehicle (VEH) or GRP (1000 nM) for 4 h. Expressions of GLP-1, GRPR, NFAT5, and proglucagon in L-cells were measured by immunoblotting and RT-qPCR. (**A**), 1 set of blots from 3 independent experiments. (**B**,**D**,**F**), quantifications of GLP-1, GRPR, and NFAT5 protein levels in L-cells after 4-h treatment. GAPDH or tubulin protein was used for normalization of the data. * *p* < 0.05 vs. 90 mM or 145 mM. ^#^ *p* < 0.05 vs. 145 mM. One-way ANOVA, Holm–Sidak test, *n* = 3/group. (**C**,**E**,**G**), quantifications of proglucagon, GRPR, and NFAT mRNA in L-cells after treatment for 4 h. GAPDH or tubulin mRNA was used for normalization of the data. Quantifications were finally expressed as fold-change, relative to the 90 mM group. * *p* < 0.05 vs. 90 mM or 145 mM. ^#^ *p* < 0.05 vs. 145 mM. One-way ANOVA, Holm–Sidak test, *n* = 3/group.

GRP exerts its effects by binding to the gastrin-releasing peptide receptor (GRPR), a member of the G protein-coupled receptor (GPCR) superfamily that is expressed in the endocrine, GIT, nervous, and respiratory systems, among others [20,21]. To determine the role of GRPR in GLP-1 secretion induced by an increase in NaCl concentration in L-cells, we quantified the mRNA and protein expressions of GRPR in L-cells treated with varying concentrations of NaCl. The protein expression of GLP-1 was increased (1.5-fold) by 170 mM NaCl, relative to 90 mM or 145 mM, but was not affected by 1000 nM GRP (Figure 3A,B), results that do not mimic the GLP-1 secreted into the incubation medium (Figure 2) and could be taken to indicate that GRP affects the release but not synthesis of GLP-1. The mRNA for proglucagon, which codes for a precursor protein for GLP-1 expressed in L-cells was similarly increased (1.5-fold) by 170 mM NaCl medium, relative to 90 mM and 145 mM NaCl (Figure 3C); GRP, had also no effect on proglucagon mRNA, regardless of sodium concentration.

GRPR protein (Figure 3A,D), as well as GRPR mRNA (Figure 3E), was increased in the medium containing either 90 mM (1.5-fold) or 170 mM NaCl (1.7-fold), relative to 145 mM NaCl. However, the presence of GRP in the incubation media did not change GRPR expression (Figure 3A,D,E). Since NFAT5 expression is increased by hypertonicity [22], we quantified the mRNA and protein expression of NFAT5 in media in response to increasing sodium concentrations. Similar to the GRPR results, the mRNA and protein expressions of NFAT5 were increased in the medium containing either 90 mM (1.7-fold) or 170 (2-fold) NaCl, relative to 145 mM NaCl. The presence of GRP, also, did not change NFAT5 expression (Figure 3A,F,G). These results suggest that low or high NaCl concentration can stimulate the expression of GRPR and NFAT5 in L-cells. These effects could not be related to differences in osmolality because osmolality was kept at 340 mOsm/L with mannitol.

### 2.3. The Effect of Sodium and GRP on GLP-1 Secretion and Expressions of GRPR and NFAT5 in Mouse Ileum

**Figure 4:**
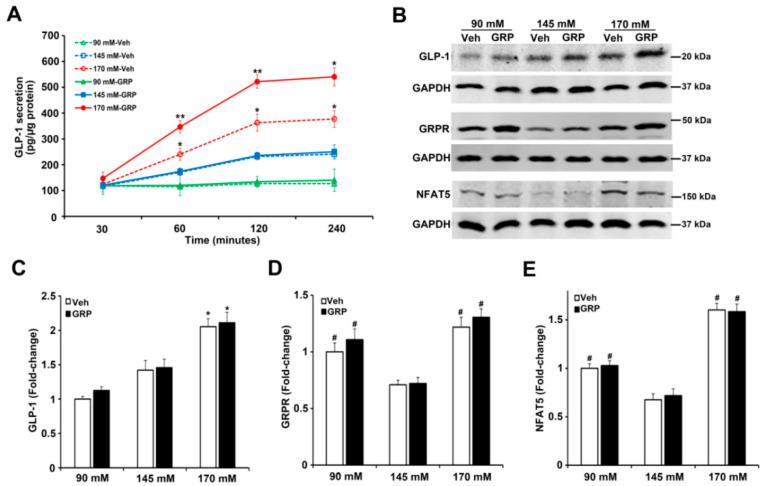
Effect of different concentrations of sodium and GRP on GLP-1 secretion and expressions of GLP-1, GRPR, and NFAT5 in ileum from BALB/c mice. (**A**) The effect of 90 mM, 145 mM, and 170 mM NaCl on GLP-1 secretion in ileal slices. The ileal slices were exposed to 90 mM, 145 mM, or 170 mM NaCl, and treated with 1000 nM GRP at 30, 60, 120, and 240 min. *n* = 3/group, * p < 0.05 ** *p* < 0.01 vs. all others at the same time point, Two-way ANOVA (F = 14.56; *p* < 0.0001), followed by a Bonferroni multiple comparison test, *n* = 5/treatment. (**B**) At the end of the treatment, the tissues were collected and subjected to immunoblotting for GLP-1, GRPR, and NFAT5 protein. One set of blots from three independent experiments is shown. (**C**–**E**) quantifications of GLP-1,GRPR, and NFAT5 protein in ileal slices after treatment for 4 h. GAPDH protein was used for normalization of the data. Quantifications were finally expressed as fold-change, relative to the 90 mM NaCl. *n* = 3/group, * *p* < 0.05 vs. 90 mM or 145 mM. ^#^ *p* < 0.05 vs. 145 mM. One-way ANOVA, Holm–Sidak test. VEH = vehicle.

GLP-1 is primarily released from the intestinal endocrine L-cells, which are located mainly in the ileum [6,9,11]. In order to study further the effect of sodium on GLP-1 secretion in the absence of non-ileum-related mechanisms, e.g., nervous system, or mechanical distension [11], pieces of mouse ileum were exposed ex vivo to media containing different concentrations of NaCl (90 mM, 145 mM or 170 mM) for 30’, 1 h, 2 h, and 4 h, in the presence of vehicle or GRP (1000 nM) and measured GLP-1 secretion into the incubation medium. As shown in Figure 4A, ileal GLP-1 secretion increased with time in a sodium concentration-dependent manner, similar to those observed in human L-cells (Figure 2A,B). The presence of GRP in the incubation medium enhanced the response, i.e., an increase in GLP-1 secretion, only in ileal slices incubated in the highest NaCl (170 mM) concentration (Figure 4A). These results are consistent with GLP-1 secreted from human L-cells in response to sodium and GRP (Figure 2A,B). Furthermore, similar to the results obtained with L-cells, the protein expression of GLP-1 in mouse ileum was increased only by 170 mM NaCl, relative to 90 mM or 145 mM (2.1-fold or 1.4-fold, respectively) and not affected by GRP (Figure 4B,C). Similar to the results in L-cells, GRPR protein (Figure 3A,B), as well as GRPR mRNA (Figure 3C), in mouse ileum was increased (1.5-fold or 1.8-fold) in medium containing either 90 mM or 170 mM, respectively, relative to 145 mM NaCl (Figure 4B,D). However, the presence of GRP in the incubation media did not change GRPR expression (Figure 4B,D), similar to that found in human L-cells. NFAT5 protein level was increased (1.5-fold or 2.4-fold) in medium containing either 90 mM or 170 mM NaCl, relative to 145 mM NaCl; the presence of GRP, also, did not change NFAT5 expression (Figure 4B,E), again similar to that found in human L-cells.

### 2.4. NFAT5 Is Involved in the High NaCl-Induced GLP-1 Secretion Caused by GRP

**Figure 5:**
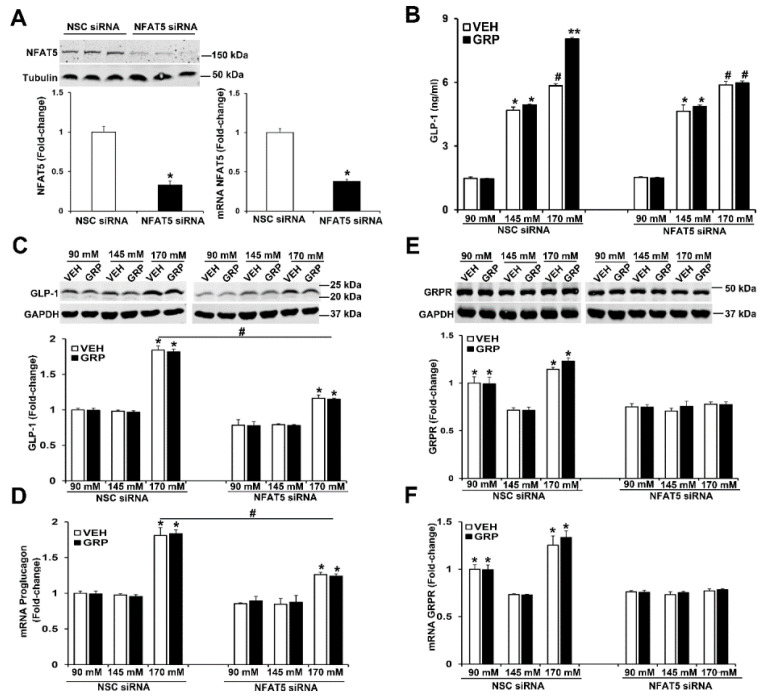
Silencing NFAT5 minimizes the high sodium-induced increase in GLP-1 secretion and blocks the sodium-induced increase in GRPR expression in human L-cells. (**A**) The same quantity of L-cells was transfected with NFAT5 siRNA or non-silencing (NSC) siRNA. Forty-eight hours after transfection, NFAT5 protein or NFAT5 mRNA was quantified by immunoblotting or RT-qPCR, respectively. Tubulin was used for normalization of the data. Quantifications were finally expressed as fold-change over the NSC siRNA group. *n* = 3/group, * p < 0.05 vs. NSC siRNA, *t*-test (**B**) the secretion of GLP-1 was quantified by ELISA in the incubation medium of L-cells. *n* = 3/group, * p < 0.05 vs. 90 mM. *^#^ p* < 0.05 vs. 90 mM or 145 mM, ** p < 0.05 vs. others. (**C**) Protein or (**D**) mRNA expression of GLP-1 in human L-cells was quantified by immunoblotting or RT-qPCR, respectively. GAPDH was used for normalization of the data. Quantifications were finally expressed as fold-change over the NSC siRNA 90 mM group. *n* = 3/group. * p < 0.05 vs. 90 mM or 145 mM. *^#^ p* < 0.05 NSC siRNA 170 mM vs. NFAT5 siRNA 170 mM. (**E**) Protein or (**F**) mRNA expression of GRPR in human L-cells was quantified by immunoblotting or by RT-qPCR, respectively. GAPDH was used for normalization of the data. Quantifications were finally expressed as fold-change over the NSC siRNA 90 mM group. *n* = 3/group. * p < 0.05 vs. 145 mM. One-way ANOVA, Holm–Sidak test. VEH = vehicle.

As aforementioned, NFAT5 expression is increased by tonicity [22]. We studied the role of NFAT5 on GLP-1 secretion and expression of GLP-1 and GRPR in L-cells by downregulating NFAT5 expression. The mRNA and protein expressions of NFAT5 were decreased by 60% in human L-cells transfected with NFAT5 siRNA, relative to cells transfected with non-silencing (NSC) siRNA (Figure 5A). Two days after transfection, the human L-cells were exposed to media with 90 mM, 145 mM, and 170 mM NaCl, (all osmolalities adjusted to 340 mOsm/L of mannitol) and treated with vehicle or 1000 nM GRP for 4 h. Similar to what was observed in non-transfected human L-cells (Figure 2A,B and Figure 3A), in those cells transfected with non-silencing (NSC) siRNA, GLP-1 secretion was increased by NaCl in a concentration-dependent manner (Figure 5B). Only in the high sodium concentration (170 mM NaCl) did GRP enhance the secretion (Figure 5B) of GLP-1 in NSC-transfected cells. In contrast to the ability of GRP to increase GLP-1 secretion, GRP had no effect on GLP-1 (proglucagon) mRNA and protein expression (Figure 5C,D), similar to those shown in human L-cells (Figure 3A–C) and slices of mouse ileum (Figure 4B,C), indicating that GRP affects the release but not the synthesis of GLP-1.

The silencing of NFAT5 prevented the ability of GRP to enhance GLP-1 secretion under high sodium (170 mM NaCl) concentration (Figure 5B). In addition, the silencing of NFAT5 impaired the ability of high sodium (170 mM NaCl) to increase GLP-1 protein and mRNA (proglucagon) (Figure 5C,D) and prevented the ability of low sodium and high sodium to increase GRPR protein and mRNA expressions (Figure 5E,F). These results indicated that NFAT5 is necessary for the GRP-mediated enhancement of GLP-1 secretion under high sodium concentration. In addition, NFAT5 is needed for 90 mM and 170 mM sodium to increase GRPR mRNA and protein in human L-cells (Figure 5E,F). By contrast, GRP did not affect GRPR expression. These results suggest that under high salt condition, NFAT5 is required for GRP to stimulate GLP-1 secretion, but NFAT5, not GRP, increases GRPR transcription/translation. These studies also show that the ability of normal sodium concentration to increase GLP-1 secretion is independent of NFAT5 and GRPR, the mechanism of which remains to be determined.

### 2.5. Sodium-Induced Binding of NFAT5 to GRPR Gene Promoter Increases Its Transcription and Subsequent Translation

**Figure 6:**
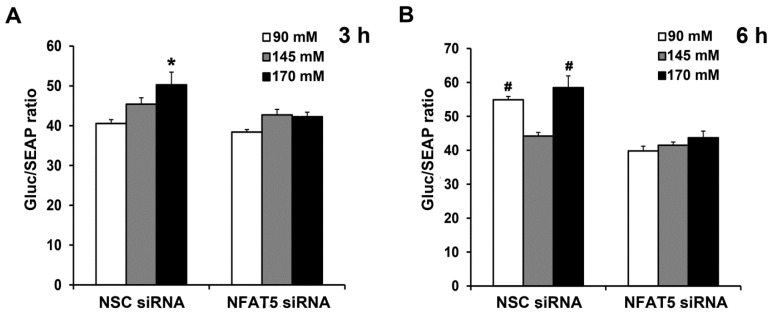
Sodium-induced increase in NFAT5 expression occurs via the *GRPR* promoter. The same quantity of human L-cells was co-transfected with the NFAT5 siRNA or non-silencing (NSC) siRNA and promoter reporter clone for human GRPR. After 48 h, the cells were exposed to fresh medium with varying concentrations NaCl. Gluc (*Gaussia luciferease*) and SEAP (Secreted Alkaline Phosphatase) activities were determined using Secrete-Pair Dual Luminescence Assay Kit at 3 h (**A**) and 6 h (**B**). Results were normalized with corresponding SEAP activity. *n* = 3/group. * p < 0.05 vs. others. *^#^ p* < 0.05 vs. NSC siRNA 145 mM or NFAT5 siRNA. One-way ANOVA, Holm–Sidak test.

Next, we determined if sodium induces the binding of NFAT5 to GRPR gene promoter, using a dual luminescence assay. We generated a reporter of GRPR transcription consisting of a portion of the GRPR promoter that included the potential NFAT5 site fused to luciferase. We used the luminescence readout from the reporter to determine the potential contribution of this site to levels of GRPR transcription by downregulating NFAT5 expression. Two days after co-transfection of the human L-cells with NFAT5 siRNA or non-silencing (NSC) siRNA and promoter reporter clone for human GRPR, the human L-cells were treated for 3 h or 6 h with different concentrations of NaCl in the medium. The Gluc/SEAP activity in NSC siRNA-treated cells was increased by 170 mM NaCl; the stimulatory effect was prevented by the silencing of NFAT5 (Figure 6A). Six hours after NaCl treatment, both Gluc/SEAP activities were increased in NSC siRNA-90 mM and NSC siRNA-170 mM; the increase was prevented by silencing of NFAT5 (Figure 6B). These results confirmed the role of NFAT5 in the high sodium-mediated increase in GRPR transcription and translation. Surprisingly, low sodium concentration also increased GRPR promoter activity, via NFAT5.

### 2.6. Gastrin, GLP-1, or Gastrin plus GLP-1 Negatively Regulates the Sodium Transporter/Exchanger/Pump in Human Renal Proximal Tubule Cells (hRPTCs)

**Figure 7:**
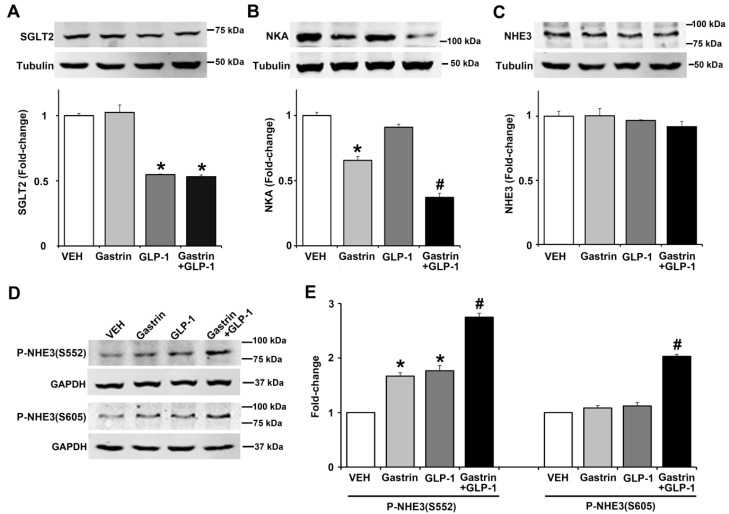
Effect of gastrin and GLP-1 on sodium transporter/pump/exchanger in human renal proximal tubule cells (hRPTCs). The hRPTCs were treated with gastrin (100 nM), GLP-1 (10 nM), or gastrin plus GLP-1 for 3 h. (**A**) SGLT2 protein, quantified by immunoblotting, was normalized by tubulin *n* = 3/group. * p < 0.05 vs. VEH (vehicle)- or gastrin-treated group. (**B**) NKA (Na^+^-K^+^/ATPase) protein, quantified by immunoblotting, was normalized by tubulin. *n* = 3/group. * p < 0.05 vs. VEH or GLP-1 group. *^#^ p* < 0.05 vs. gastrin-treated group. (**C**) Total NHE3 protein, quantified by immunoblotting, was normalized by tubulin. *n* = 3/group, no differences among the groups. (**D**) Immunoblots of phospho-NHE3. 1 set of blots from three independent experiments is shown. (**E**) Phospho-NHE3 (SEs of VEH are too small to show), quantified by immunoblotting, was normalized by GAPDH. *n* = 3/group. * p < 0.05 vs. VEH group. *^#^ p* < 0.05 vs. others. One-way ANOVA, Holm–Sidak test.

Since gastrin [3,4] or GLP-1 [6,7,23] can decrease renal sodium transport, we studied the effect of gastrin (100 nM), GLP-1 (10 nM), or gastrin plus GLP-1 on SGLT2, Na^+^-K^+^/ATPase, and NHE3 expressions, as well as NHE3 phosphorylation in human renal proximal tubule cells (hRPTCs). After 3 h of incubation, the expression of SGLT2 was inhibited (by 45%) by GLP-1 but not by gastrin which also did not affect the inhibitory effect of GLP-1 (Figure 7A). By contrast, gastrin, but not GLP-1, decreased Na^+^-K^+^/ATPase expression by 34%, although GLP-1 potentiated the inhibitory effect of gastrin (Figure 7B). We have reported that gastrin decreases Na^+^-K^+^/ATPase expression in the mouse kidney and hRPTCs [24]. Several hormones, including gastrin [25] and GLP-1 [24], induce natriuresis by inhibiting NHE3, located at the apical membrane of the renal proximal tubule [25]. The phosphorylation of NHE3 at serine 661 increases NHE3 activity, whereas the phosphorylation of NHE3 at serines 552 and 605 inhibits NHE3 activity, facilitating natriuresis [25]. Consistent with our report [25], gastrin did not affect the expression of total NHE3, as did GLP-1, by itself or in combination with gastrin (Figure 7C). However, gastrin and GLP-1 independently increased the phosphorylation of NHE3 at serine 552 by 1.7-fold and 1.8-fold, an effect that was greater (2.7-fold) with the combination of gastrin and GLP-1 (Figure 7D,E). Neither gastrin nor GLP-1 treatment increased the phosphorylation of NHE3 at serine 605, but their combination did, by 1.8-fold, relatively to gastrin or GLP-1 treatment group (Figure 7E). These results show that the effects of GLP-1 and gastrin on renal proximal tubule sodium transport are not the same but may complement each other.

## 3. Discussion

Organ-to-organ communication is important in the maintenance of normal fluid and electrolyte balance and blood pressure (BP) [1]. One such organ-to-organ communication involves the GIT and kidney. The balance between intestinal absorption and urinary excretion of sodium is essential for the maintenance of sodium homeostasis in the body [2,3,5]. Sensing the amount of ingested sodium by the GIT may be an important mechanism by which sodium balance is regulated [3,4,5]. GIT-derived hormones and peptides participate in the regulation of renal sodium transport and blood pressure [3,5,6,7]. We and others have reported that gastrin, secreted by G-cells in the stomach and duodenum of salt-resistant mice, is involved in the regulation of sodium balance and BP [3,4,5]. Gastrin secreted by G-cells in the stomach and duodenum, in response to high salt diet, acts on the gastrin receptor, CCKBR, in the kidney to increase sodium excretion [3,4,5]. This occurs by the inhibition of the activities of renal tubular NHE3 and Na^+^, K^+^-ATPase [24,25], which is aided by dopamine produced in the kidney [4,26].

An apparent contradiction of the importance of the gastro-renal axis in the maintenance of normal sodium and fluid balance and blood pressure is the observation that the blood pressure is not increased in patients who have had gastric bypass, which should affect gastrin secretion by the stomach. Actually, high blood pressure is normalized by gastric bypass in 38% in adults (Roux-en-Y gastric bypass) and 74% in adolescents (Roux-en-Y gastric bypass and sleeve gastrectomy) with hypertension [27,28]. Roux-en-Y gastric bypass in rats enhances their ability to excrete a sodium load [29]. As it turns out, sleeve gastrectomy actually increases plasma gastrin levels after a mixed meal [30]. By contrast, Roux-en-Y gastric bypass prevents the increase in plasma gastrin after a mixed meal but either type of bypass increases plasma GLP-1 [30,31,32].

GLP-1 is another gut hormone that may also participate in the regulation of sodium balance and BP [23,32]. GLP-1 is mainly released from the L-cells of small intestines in response to hormonal, neural, and nutrient stimuli [5,6,7,8]. The neuroendocrine system plays an important role in stimulating GLP-1 secretion, especially the initial rapid rise in GLP-1 secretion after the ingestion of food [7,8,9,13]. Glucose-induced GLP-1 secretion is sodium-dependent [13]. The increase in intracellular calcium needed for GLP-1 secretion is also due to sodium-dependent cell excitability [8,13]. However, the effect of varying concentrations of sodium on GLP-1 secretion and its potential mechanism have not been reported.

We explored the effect of varying concentrations of sodium on GLP-1 secretion in vivo, ex vivo, and in vitro. After gavage with HS diet, the serum GLP-1 levels of salt-resistant BALB/c mice were higher than the GLP-1 levels after gavage with NS or LS diet. GLP-1 secretion after a meal is biphasic [9,33,34]. The initial peak usually occurs 15–30 min after a meal. There is a proximal-distal neuroendocrine loop mechanism underlying the regulation of GLP-1 secretion at the first peak. After a meal, nutrients in the duodenum activate this neuroendocrine loop by stimulating the release of GRP from enteric neurons. The released GRP stimulates GLP-1 secretion from L-cells in the ileum and colon. The second peak, which is less than the first peak, occurs 30–60 min after a meal and is caused by direct stimulation of intestinal L-cells [33,34,35]. Our in vivo study also showed that GLP-1 secretion caused by sodium is somewhat biphasic after a meal, especially that in response to the diet containing HS.

GRP, a neuropeptide distributed widely in the GIT, also stimulates GLP-1 secretion in L-cells [9,10,12]. GRP antagonism prevents the increase in fat-induced GLP-1 secretion [9]. The increase in GLP-1 secretion in response to gastric glucose is impaired in GRPR-deficient mice [10]. As aforementioned, the ability of nonmetabolizable sugars to increase GLP-1 secretion by the ileum is sodium dependent, involving sodium-glucose cotransporters [8,9,13,35]. GRP can increase sodium entry into the cell by stimulating Na^+^-K^+^/ATPase activity, which could be related to its stimulatory effect of GLP-1 secretion [12]. To determine the direct effect of sodium on the secretion of GLP-1, we studied human L-cells, which have been reported to be a good model to study the cellular mechanisms involved in the secretion of GLP-1 [19]. We found that GLP-1 secretion in L-cells was sodium concentration- and time-dependent. In addition, high concentrations of GRP (500 and 1000 nmol/L) potentiated the stimulatory effect of 170 mM sodium on GLP-1 secretion. We did not measure the amount of GRP in the stomach or intestines in response to gavage of high sodium diet or in L-cells exposed to varying concentrations of sodium. In C57Bl/6-KsJ mice on a regular diet, basal GRP concentration in small intestines is 44 nM [36]. However, in Wistar rats, the duodenal and ileal GRP concentrations are 96 nM and 50 nM, respectively [37]. It is, therefore, possible that the lowest concentration of GRP, i.e., 500 nM that increased GLP-1 secretion in L-cells could be attained in vivo. However, our studies were not meant to determine the effect of GRP on GLP-1 secretion, which is known [9], but rather the effect of sodium and gastrin on GLP-1 secretion. To prove that the stimulatory effect of sodium on GLP-1 secretion occurs in tissues containing normal L-cells, we studied the effect of varying concentrations of sodium on GLP-1 secretion in slices of ileum from salt-resistant BALB/c mice [38]. In these ileal slices, 145- and 170-mM sodium but not 90 mM sodium increased GLP-1 secretion, peaking at 120 min, remaining at the same level at 240 min. Similar to the human L-cell studies, GRP potentiated the stimulatory effect of 170 mM sodium at 60, 120, and 240 min. Thus, sodium can stimulate GLP-1 secretion in isolated and intact L-cells, in a concentration-dependent manner, and GRP enhances the high sodium-induced GLP-1 secretion.

NFAT5 (TonEBP/OREBP), a member of the Rel-family of transcription factors, protects cells from hypertonic stress [15,39]. NFAT5, which is expressed in almost all tissues in the body [17,39], has important functions besides those related to hypertonicity [16,39]. NFAT5 can also be regulated by stimuli independent of tonicity, including glucose, calcium, and sodium chloride/superoxide [15,16,17]. Since NFAT5 expression is increased by tonicity [22,39] and bombesin, a GRP analogue, can activate NFATc1, we studied, ex vivo, the expression of GRPR and NFAT5 in mice ileum treated with different concentrations sodium with or without GRP. GLP-1 expression in the ileum was increased by 145 mM and 170 mM sodium. GRPR and NFAT5 expressions were also increased by 170 mM sodium.

Surprisingly, 90 mM sodium also increased the NFAT5 and GRPR expression in the mouse ileum. These results were confirmed in human L-cells. In these human L-cells, the low and high sodium concentrations increased the expression of NFAT5 and GRPR at the mRNA and protein levels. The ability of low sodium concentration would seem to contradict the accepted role of NFAT5 in protecting cells from hypertonic stress [15,39]. However, in our studies, the osmolality of low and normal sodium was kept by mannitol at 340 mOsm/L to be similar to that with 170 mM sodium. The reason for the stimulatory effect of low and high sodium concentration, with the same osmolarity is unclear, but low sodium concentration can regulate gene transcription by epigenetic mechanisms [40].

We concentrated our subsequent studies on the role of NFAT5 in the increase in GLP-1 secretion, and protein and mRNA expressions induced by high sodium concentration. The siRNA-induced silencing of NFAT5 in human L-cells blocked the increase in GLP-1 secretion by 1000 nM GRP in the presence of high sodium (170 mM) concentration. NFAT5 siRNA also partially reduced the increase in GLP-1 expression (mRNA and protein levels), induced by 170 mM sodium. However, GRP did not affect GLP-1 mRNA and protein expressions (mRNA and protein levels), at any sodium concentration, unlike the increase in GLP-1 secretion in human L-cells exposed to high sodium (170 mM) concentration. Nevertheless, NFAT5 siRNA prevented the increase in GRPR expressions (mRNA and protein) caused by low (90 mM) and high (170 mM) sodium concentration. These data indicate that the activation of NFAT5, in response to high and low sodium concentration, can regulate GRPR and partially regulate GLP-1 expression in the cell. A dual luminescence assay verified that sodium increased GRPR expression in human L-cells that was abrogated by NFAT5 silencing with siRNA, related to its binding to GRPR gene promoter. However, in the current study, both LS and HS stimulated NFAT5 and subsequently GRPR. By contrast, GRP stimulated GLP-1 secretion only when human L-cell were exposed to HS. A certain amount of sodium may be necessary for GLP-1 secretion [8,9]. Thus, GRP is downstream of NFAT5, but GLP-1 secretion and expression can be GRP-independent. GLP-1 levels can be affected not only by its synthesis but also by its degradation, such as that exerted by dipeptidyl peptidase-4 (DPP-4). Although some of the effects of DDP-4 inhibitors may be related to an increase in GLP-1 levels, DPP-4 cleaves many other peptide hormones, including brain/atrial natriuretic peptide, neuropeptide Y, peptide YY, and stromal-derived factor 1 [41]. There are no reports of a direct effect of DDP-4 on the activities of NHE3 or Na^+^-K^+^/ATPase in the kidney.

Gastrin inhibits sodium transport in hRPTCs and rodent kidney [4,24,25,26]. The inhibitory effect of GLP-1 on renal proximal tubule sodium transport in rodents and humans has also been reported [6,23,42]. We studied the effect of GLP-1, by itself, or in the presence of gastrin in hRPTCs. In hRPTCs, GLP-1 decreased the expression of SGLT2; gastrin, by itself, had no effect and did not affect the inhibitory action of GLP-1. This inhibitory effect of GLP-1 on renal SGLT2 is consistent with studies showing that the combination of a GLP-1 agonist and an SGLT2-inhibitor has additive effects on lowering HbA1c and systolic blood pressure, body weight, and cardiac risk [43]. GLP-1, which by itself, had no effect on Na^+^-K^+^/ATPase expression in hRPTCs, potentiated the inhibitory effect of gastrin. Neither GLP-1 nor gastrin affected total NHE expression. Phosphorylation of NHE3 can decrease its activity [23,25]. Either GLP-1 or gastrin increased the phosphorylation of NHE3 at serine 552; their combination increased further the phosphorylation at serine 552. Neither gastrin nor GLP-1 affected the phosphorylation of NHE3 at serine 605, but their combination did. Thus, the ability of GLP-1 to inhibit renal sodium transport may be complemented by gastrin. The ability of GLP-1 to inhibit renal sodium transport persists in diabetes. The GLP-1 analogue, exendin 4, has been reported to decrease Na^+^-K^+^/ATPase activity in renal distal tubule cells of non-diabetic and diabetic mice [44]. Lixisenatide, a short-acting GLP-1 receptor agonist, can also increase sodium excretion by direct inhibition of NHE3 in the renal proximal tubule in humans with type 2 diabetes [45].

**Figure 8:**
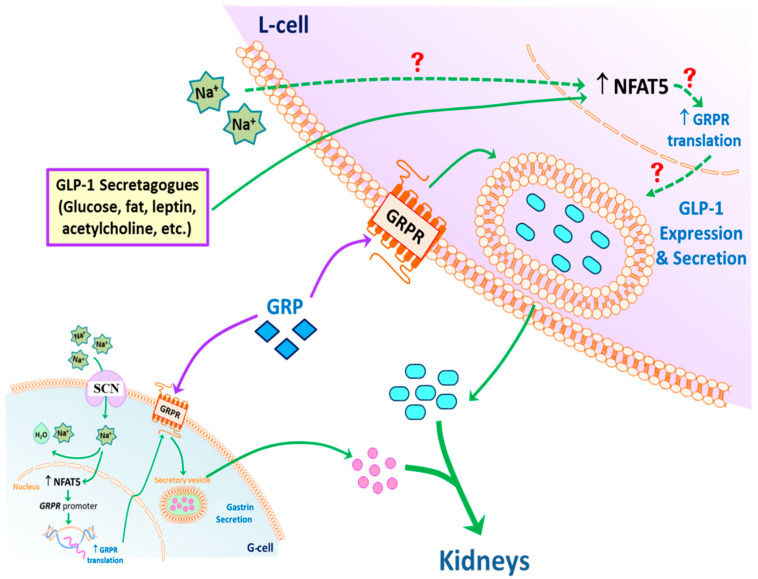
Potential mechanism of GLP-1 secretion. An increase in intracellular Na^+^, via SCN, in L-cells (and possibly G-cells) stimulates the expression of NFAT5, which increases the translation of glucagon-like peptide-1 (GLP-1). NFAT5 may also increase the secretion of gastrin releasing peptide (GRP) and transcription of GRP receptor (GRPR). High concentrations of GRP may potentiate the ability of sodium to increase GLP-1 secretion. The secreted GLP-1, together with gastrin, decreases renal sodium transport. This effect is facilitated by dopamine, acting via D_1_-like receptors (D_1_R and D_5_R) to increase sodium excretion, especially during moderate volume expansion. Exposure of L-cells to low intracellular Na^+^ also increases the expression of NFAT5 but does not stimulate GLP-1 secretion, the cause of which remains to be determined.

Based on the results of the current studies, we propose the following model of GLP-1 secretion in response to high sodium concentration that reaches the ileum and colon after a high salt diet (Figure 8). GLP-1 secretion by L-cells can be directly stimulated by sodium. NFAT5 expression in L-cells can also be stimulated by high sodium concentration. NFAT5 binds to the promoter of GRPR, increasing the transcription and subsequent translation of GRPR. The endogenous GRP acting on the increased expression of GRPR can facilitate the release of GLP-1 at high sodium concentrations. In addition, circulating GLP-1 is taken up by the RPT from the circulation (not shown), and with gastrin decreases renal sodium transport, to maintain sodium balance and normal blood pressure in health and disease. Not shown is the fact that GRP can also increase GLP-1 secretion by increasing gastrin secretion, which can also increase the release of GLP-1 [46].

## 4. Materials and Methods

### 4.1. Cell Culture and Reagents

The NCI-H716 intestinal cell line (ATCC^®^ CCL-251™, Manassas, VA, USA) (L-cells), originally obtained from ascitic fluid of a 33-year-old Caucasian male with poorly differentiated adenocarcinoma of the colon, was used to study the regulation of GLP-1 expression and secretion [19]. The L-cells were grown in suspension in RPMI 1640 (ATCC, #30-2001, Manassas, VA, USA), supplemented with 10% FBS, 100 IU/mL penicillin, and 100 µg/mL streptomycin in a 37 °C incubator with 5% CO_2_ and 95% humidity. Penicillin, streptomycin, and FBS were from Thermo Fisher Scientific (Waltham, MS, USA). GRP (#AS-24213 AnaSpec Inc., Fremont, CA, USA) was dissolved in sterile deionized water. Immortalized hRPTCs [47], isolated from a normotensive Caucasian male, were grown in DMEM/F-12, supplemented with 2% fetal bovine serum, containing epidermal growth factor (10 ng/mL), insulin, transferrin, and selenium cocktail (5 µg/mL each), and dexamethasone (4 ng/mL), in a 37 °C incubator with 5% CO_2_ and 95% humidity.

The primary antibodies were: rabbit polyclonal anti-GRPR (1:1000, #PA5-27073); rabbit polyclonal anti-GLP-1 (1:500, #710320) antibodies (Thermo Fisher Scientific, Waltham, MS, USA), rabbit polyclonal anti-NFAT5 (1:1000, GTX110903); mouse monoclonal anti-GAPDH (1:10,000, GTX627408) antibodies (Genetex, Irvine, CA, USA), and mouse monoclonal anti-α-tubulin (1:10,000, T5168) antibodies (Sigma-Aldrich, St. Louis, MO, USA). The corresponding secondary antibodies were IRDye^®^ 800CW or 680RD (LI-COR, Lincoln, NE, USA).

The primary antibodies for renal sodium transport were: rabbit polyclonal SGLT2 antibodies (1:1000, ab37296; Abcam, Cambridge, MA, USA); rabbit polyclonal NHE3 antibodies (1:1000, MP635E; GenWay BioTech Inc., San Diego, CA, USA), mouse monoclonal Na^+^-K^+^/ATPase antibodies (1:1000, 05-369; Millipore Sigma, Burlington, MA, USA); and mouse monoclonal p-serine552-NHE3 and p-serine605-NHE3 antibodies (1:200, sc-53962 and sc-53961, Santa Cruz Biotechnology, Dallas, TX, USA).

### 4.2. GLP-1 Secretion

#### 4.2.1. In Vivo Studies

BALB/c mice (The Jackson Laboratory, Bar Harbor, ME, USA) were fed rodent chow containing 0.8% NaCl. Food was withheld overnight and the following morning, blood (100–120 µL) was drawn from the caudal vein. One hour later, the mice were gavaged with 1 mL of low (LS), normal (NS), or high salt (HS) food. LS, NS, and HS food contained <0.04 mmol sodium, 0.56 mmol sodium, or 0.84 mmol sodium/0.1 kg body weight, respectively. Post gavage, blood was drawn from the caudal vein at 15 min, 30 min, and 60 min. GLP-1 levels were measured by SimpleStep ELISA^®^ Kit (ab184857, Abcam, Cambridge, MA, USA).

#### 4.2.2. In Vitro Studies

Equal numbers of NCI-H716 intestinal (CCL-251) cells (human L-cells) at the density of 2 × 10^6^ cells/mL in six-well plates were treated with 1 mL fresh medium containing varying concentrations of sodium as NaCl (90, 145, and 170 mM); the osmolarities of the 90 and 145 mM NaCl were adjusted to 340 mOsM [38] with mannitol. At each sodium concentration, the cells were treated with vehicle or two concentrations of GRP (500 and 1000 nM). The plates were incubated in 37 °C incubator with 5% CO_2_ and 95% humidity with constant shaking, for 1 h, 2 h, 3 h, and 4 h. Equal volume (100 μL) of supernatants were collected at each time point and stored at −20 °C until used for assays. At the 4 h-time point, the cells were collected for protein and RNA extraction. Protein concentrations were assayed by bicinchoninic acid (BCA).

Equal numbers of hRPTCs, serum-starved for 3 h, were treated with gastrin (100 nM) [24], GLP-1 (10 nM) [48], or gastrin (100 nM) plus GLP-1 (10 nM) for 3 h. Gastrin (human gastrin-1, RP12740; GenScript, Piscataway, NJ, USA) and GLP-1 (human GLP-1 (7–36), RP10773; GenScript, Piscataway, NJ, USA) were dissolved in sterile-deionized water.

#### 4.2.3. Ex Vivo Studies

Freshly-harvested distal ileums of mice fed normal NaCl (0.8%) diet were sliced into thin pieces and exposed to varying concentrations of NaCl (90, 145, or 170 mM, osmolarity adjusted with mannitol as described above), for 30 min, 1 h, 2 h, and 4 h in the absence or presence of GRP (1000 nM). After centrifugation (100× *g*, for 5 min), equal volumes of supernatant were collected and stored at −20 °C until assay of GLP-1. At the 4-h time point, the pelleted tissues were collected for protein and RNA extraction.

### 4.3. Immunoblotting

The cells or tissue samples were homogenized and lysed in 1× radioimmunoprecipitation assay buffer with protease and phosphatase inhibitors, and then sonicated for 30 s. Protein concentrations in were quantified by BCA assay kit (Cat # 23225, Pierce Biotechnology, Rockford, IL, USA). Equal amounts of protein were loaded onto 8–12% tris-glycine gels for SDS-PAGE, transferred onto nitrocellulose membranes, and then probed with primary antibody, overnight at 4 °C. The blots were then incubated with the appropriate secondary antibody, labeled with fluorescent dyes for 1 h at room temperature. Then the membranes were scanned with LI-COR near-infrared imaging system (LI-COR Biosciences, Lincoln, NE, USA), and the intensities of the signals were quantified by Odyssey^®^ CLx Imaging system (LI-COR Biosciences, Lincoln, NE, USA). The densitometry values were corrected by the expression of GAPDH or tubulin and shown as percentage of the mean density of the mock (non-silencing siRNA [NSC])-treated group. The data are expressed as fold-change or as relative percentage change over the control.

### 4.4. RNA Extraction and cDNA Preparation

Total RNA was purified using the RNeasy RNA Extraction Mini kit (Qiagen, Valencia, CA, USA). RNA samples were converted into first-strand cDNA using an RT2 First Strand kit (SABiosciences-Qiagen, Germantown, MD, USA).

### 4.5. Real-Time Quantitative Polymerase Chain Reaction

Gene expression was estimated by real-time quantitative polymerase chain reaction (RT-qPCR) (ABI Prism 7900 HT, Applied Biosystems, Foster City, CA, USA). The assay used gene-specific primers (SABiosciences-Qiagen, Germantown, MD, USA) and the SYBR Green real-time polymerase chain reaction detection method. The primers were: GCG primer sequence: forward, 5′-GAAAGAACCATCAGCATGTCTG-3′; reverse, 5′-AATTCATTGCTTGGCTGGTG-3′ GRPR primer sequence: forward, 5′-CACAAACACCAGCACTGTCT-3′; reverse, 5′-CCACTGTCGATCATCTCTGTT-3′; and NFAT5 primer sequence: forward, 5′-TTCCTATTCTGGCTTCGACATC; reverse, 5′-TGGACATTGAAGGCACTACTG-3′.

### 4.6. siRNA Transfections and Assays

L-cells were plated one day before treatment. Predesigned siRNA (SI00090097, Qiagen, Germantown, MD, USA) targeting human NFAT5 mRNA or non-silencing siRNA (NSC), as control, was transfected into the L-cells, using FuGene HD transfection reagent (E2311; Promega Corporation, Madison, WI, USA). Two days following transfection, the L-cells were exposed to varying concentrations of NaCl (90, 145, and 170 mM) and treated with vehicle (control) or GRP (100 and 1000 nM) for 4 h. Then, the supernatants were collected for GLP-1 concentration measurement. The pelleted cells were harvested for protein extraction and immunoblotting, total RNA preparation, and RT-qPCR. All Stars negative control siRNA (Qiagen, Germantown, MD, USA) was used in the control group.

### 4.7. Dual Luminescence Assay (GRPR Promoter Reporter Analysis)

Secrete-pair dual luminescence assay (GeneCopoeia, Rockville, MD, USA) was used to determine whether NFAT5 directly regulates the expression of GRPR in human L-cells. The recombinant promoter reporter clone for human GRPR (HPRM46053-PG04, GeneCopoeia, Rockville, MD, USA) containing NFAT5 binding site (5′-TGGAAANYNY-3), upstream of the *Gaussia luciferase* (Gluc) gene (GeneCopoeia, Rockville, MD, USA). The plasmid GRPR/Gluc contains a 1412-base region from the human GRPR promoter [-1231 to +181 of transcription start site (TSS)] driving the expression of naturally secreted Gluc, and a secondary reporter with a CMV promoter driving the expression of Secreted Alkaline Phosphatase (SEAP), served as an internal control. The NFAT5 siRNA or non-silencing siRNA (NSC) was co-transfected with promoter reporter clone for human GRPR, using FuGene HD transfection reagent into the L-cells. Two days later, equal quantities of L-cells were exposed to varying sodium concentrations (90, 145, and 170 mM). The L-cells were centrifuged at a speed of 100× *g*/min for 5 min and equal volumes of supernatants were collected at 3 h and 6 h time points. GRPR promoter activity was measured by using Secrete-Pair Dual Luminescence Assay Kit (Cat.No.LF061, Genecopoeia, Rockville, MD, USA). Luminescence activity was measured by a microplate reader. The relative reporter activity was calculated by normalizing the Gluc activity against SEAP activity within each sample.

### 4.8. Statistics

All data are expressed as mean ± SEM. Student’s *t*-test was used to compare two groups and one- or two-way analysis of variance (ANOVA) with Holm–Sidak or Bonferroni post-hoc test for >2 groups (GraphPad Prism, La Jolla, CA, USA). *p* < 0.05 was considered statistically significant.

## 5. Conclusions

In conclusion, this study gives a new perspective on the mechanisms of GLP-1 secretion, and the direct relationship between sodium intake and GLP-1. GLP-1 secretion by L-cells can be directly stimulated by sodium. NFAT5 expression in L-cells can also be stimulated by high sodium concentration. NFAT5 binds to the promoter of GRPR, increasing the transcription and subsequent translation of GRPR. The endogenous GRP, acting on the increased expression of GRPR, facilitates the release of GLP-1 at high sodium concentrations. In addition, circulating GLP-1 is taken up by the renal proximal tubules from the circulation, and together with gastrin, decrease renal sodium transport, to maintain normal sodium balance and blood pressure. This study provides a rationale for the use of GLP-1-based agents in treatment of hypertension and other disorders that cause sodium retention. This study also provides insight into better utilization of existing GLP-1-based drugs and further GLP-1-based drug development in the treatment of hypertension.

## Data Availability

The data that support the findings of this study are available from the corresponding author upon reasonable request.
